# Rapid gene identification in sugar beet using deep sequencing of DNA from phenotypic pools selected from breeding panels

**DOI:** 10.1186/s12864-016-2566-9

**Published:** 2016-03-15

**Authors:** David Ries, Daniela Holtgräwe, Prisca Viehöver, Bernd Weisshaar

**Affiliations:** CeBiTec & Department of Biology, Bielefeld University, D-33594 Bielefeld, Germany

**Keywords:** Mapping by sequencing, Allele frequency, *R* locus, *Beta vulgaris*, Sugar beet, SNP detection, Gene identification, Phenotypic pools

## Abstract

**Background:**

The combination of bulk segregant analysis (BSA) and next generation sequencing (NGS), also known as mapping by sequencing (MBS), has been shown to significantly accelerate the identification of causal mutations for species with a reference genome sequence. The usual approach is to cross homozygous parents that differ for the monogenic trait to address, to perform deep sequencing of DNA from F2 plants pooled according to their phenotype, and subsequently to analyze the allele frequency distribution based on a marker table for the parents studied. The method has been successfully applied for EMS induced mutations as well as natural variation. Here, we show that pooling genetically diverse breeding lines according to a contrasting phenotype also allows high resolution mapping of the causal gene in a crop species. The test case was the monogenic locus causing red vs. green hypocotyl color in *Beta vulgaris* (*R* locus).

**Results:**

We determined the allele frequencies of polymorphic sequences using sequence data from two diverging phenotypic pools of 180 *B. vulgaris* accessions each. A single interval of about 31 kbp among the nine chromosomes was identified which indeed contained the causative mutation.

**Conclusions:**

By applying a variation of the mapping by sequencing approach, we demonstrated that phenotype-based pooling of diverse accessions from breeding panels and subsequent direct determination of the allele frequency distribution can be successfully applied for gene identification in a crop species. Our approach made it possible to identify a small interval around the causative gene. Sequencing of parents or individual lines was not necessary. Whenever the appropriate plant material is available, the approach described saves time compared to the generation of an F2 population. In addition, we provide clues for planning similar experiments with regard to pool size and the sequencing depth required.

**Electronic supplementary material:**

The online version of this article (doi:10.1186/s12864-016-2566-9) contains supplementary material, which is available to authorized users.

## Background

Linking a causal mutation to a phenotype by genetic mapping is one of the major tools for uncovering gene function. However, classical ‘positional gene cloning’ by forward genetics and ‘genetic walking’ has been very time-consuming [1]. Since more than 25 years, molecular markers have been used to detect allele frequencies in pools of phenotypically similar genotypes from segregating populations, thus linking position information from genetic markers to loci contributing to specific traits [2, 3]. This method, called ‘bulk segregant analysis’ (BSA), relies on the application of molecular markers which detect DNA polymorphisms between the parents of a mapping population that are closely linked to a locus relevant for a particular trait. Such markers will co-segregate with that trait, i.e. segregate together with the respective phenotype. While QTL analysis with mapping populations is usually the more precise method to identify genes controlling a trait, pooling and BSA has the advantage of avoiding the necessity to genotype every member of the population [4, 5]. With regard to the genetic and/or genomic resolution, or the size of the genomic region to be identified, BSA and genetic mapping in general depend on the number of recombination events evaluated. In addition, mapping can only be as precise as the number of available markers allows. With the advent of next generation sequencing (NGS), this drawback has fallen away almost completely [6]. Also, the number of genomes sequenced and assembled has increased considerably over the last years [7], and elaborate tools for variant calling from whole genome shotgun sequencing datasets have become available. Together, and given that sequence variation as such exists, this has allowed tremendous progress in linking genotype to phenotype as well as in uncovering gene functions by forward genetics.

By combining BSA with NGS, the process of identifying causal genes for monogenic traits or phenotypes has been accelerated dramatically [8]. Data from NGS allow reliable detection of allelic variation in the form of single sequence polymorphisms (SNPs) and small insertions and deletions (InDels), as well as the determination of the relative abundance of these alleles. Phenotypic selection of individuals from a segregating F2 population affects the allele frequency at loci linked to the phenotype. The skews introduced by this targeted selection can be revealed by genome-wide evaluation of allele frequencies in pools of individuals that were built separately for the different phenotypes of the same trait. Genomic regions that showed diverging allele frequencies for the two parental phases can then be analyzed for mutations responsible for the phenotype in question [8].

Initially, NGS was applied to BSA for the model plant *Arabidopsis thaliana* [9]. The general approach is now referred to as ‘mapping by sequencing’ (MBS, [10]), while the original bioinformatic analysis pipeline was designated *SHOREmap*. Once the biological material, a large segregating F2 population of *A. thaliana*, was produced and phenotyped, the MBS approach allowed to map a causal gene to a small genomic region within days. In fact, due to the high density of genetic markers and the availability of reference sequences for both parental genotypes, it was even possible to identify the individual nucleotide sequence aberration that caused the mutant phenotype. The initial proof-of-concept experiment identified a non-synonymous SNP in a single gene by sequencing a DNA pool derived from 500 mutant (recessive homozygous) F2 plants [9].

While the general principle of MBS is fairly straightforward, the bioinformatics to realize reliable identification of the responsible genetic interval or genome region is not. Therefore, a number of bioinformatic analysis pipelines that deal with the massive amount of short read data for DNA sequence variation detection and allele frequency determination have been developed. By using such pipelines, MBS has been successfully applied in the model species arabidopsis, rice and fruit fly [11–13]. To allow the basic principle of MBS to work, the two alleles corresponding to the phenotypic difference that is sorted into the pools need to be associated with haplophases that offer sequence variation and thereby traceable molecular markers. To introduce such traceable markers into the F2 generation, several crossing schemes have been suggested. These include crossing to an accession that displays a relatively high degree of variation like in the initial proof-of-concept experiment where variation was derived from a cross of Col-0 x L*er*-1. In addition, mutagen-induced sequence polymorphisms have been used as markers which allowed to create the F2 in essentially the same genetic background (backcross to the unmutagenized parent of the mutant collection). This strategy permitted increased sensitivity for selecting subtle phenotypic differences [10, 13]. Common to all of these approaches is the necessity to perform a dedicated crossing experiment as a basis for phenotypic selection of F2 offspring, as well as the need to sequence the parental genotype(s) in addition to the availability of a reference genome sequence. Also, extensions of the strategy have been realized in maize. Sequence-based determination of genome-wide allele frequencies was used to assess the effect of selection for ear number in an experimental population pre- and post-selection for 30 generations. This resulted in the identification of 28 loci contributing to control of number of ears per plant in maize [14]. Recently, a method designated ‘extreme-phenotype GWAS’ (XP-GWAS) was described that relies on measurement of allele frequencies in pools of individuals from a diversity panel that have extreme phenotypes. By using the kernel row number trait as an example, several linked QTL were resolved and trait-associated variants within a single gene under a QTL peak were detected [15].

For all important crop plants, breeders maintain many lines or accessions harboring important traits and phenotypes. These lines are usually phenotypically and genetically well characterized. Using pools of such plant lines (or accessions) for MBS could make, at least for some cases, time consuming crossings to generate segregating F2 populations unnecessary. Another aspect is that the number of recombination events covered by the pools subjected to BSA or MBS is a main determinant of the resolution of the mapping, or, in other words, the size of the genetic interval that should contain the causal gene [16]. Because recombination events from several generations are accumulated during the breeding history of plant lines in a breeding panel [17], the accessions from a breeding panel included in a pool contribute more recombination events than F2 plants from a single cross. This should shorten the genetic interval and allow to more precisely reveal the position of the causative gene.

**Fig. 1:**
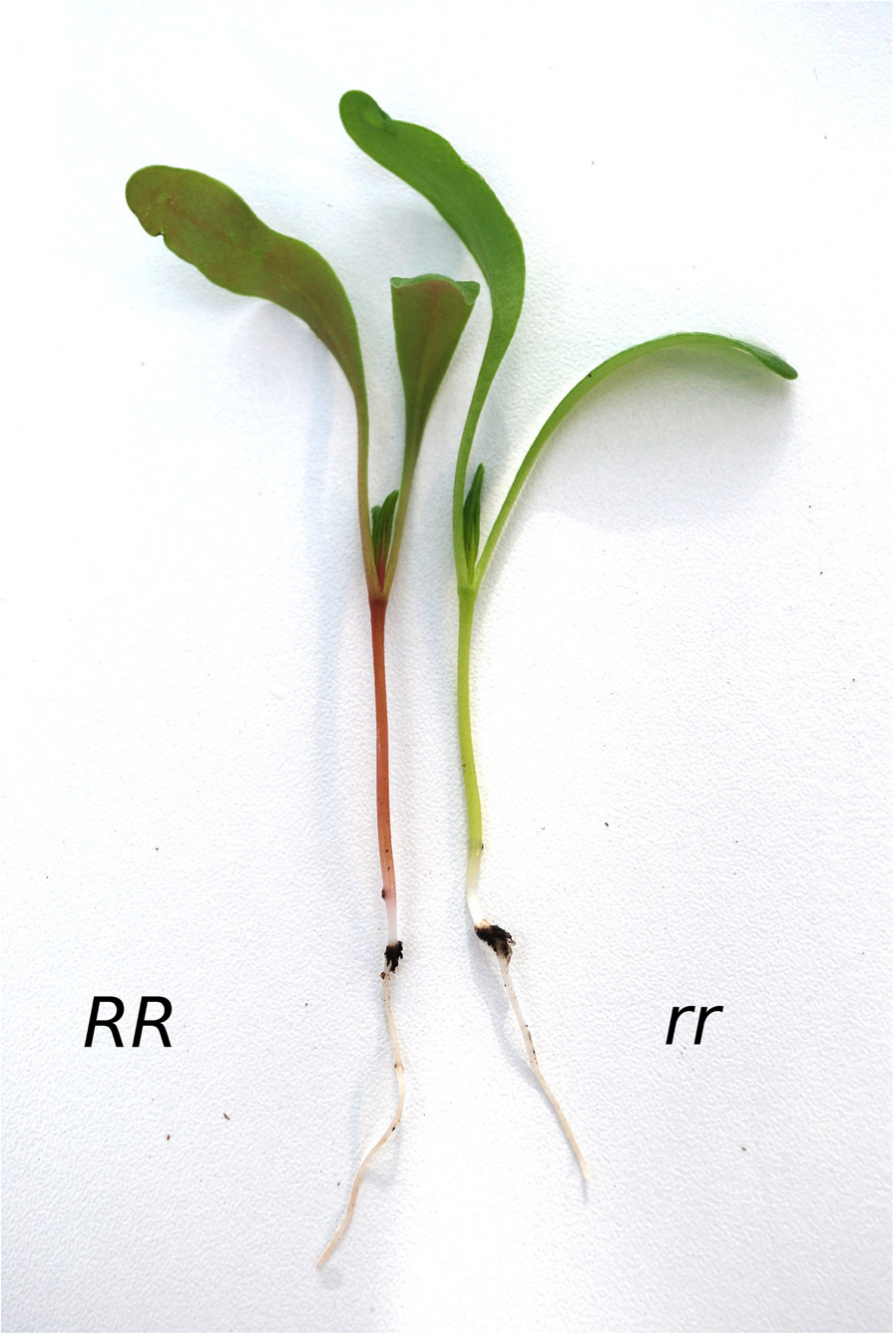
The phenotype of sugar beet (*B. vulgaris*) seedlings homozygous for a mutation in the *RED* gene. Betalain deficient plants (right) do not show any red pigmentation, the hypocotyl appears green due to the lack of betalain pigments. The two phenotypes are easy to distinguish, since homozygous plants show either a fully expressed red color in the hypocotyl, or a complete lack of it. *RR* and *rr*, genotype of the respective seedling

Sugar beet (*Beta vulgaris* ssp. *vulgaris*) is a diploid crop plant with 9 chromosomes in the haploid genome and an estimated genome size of about 730 Mbp [18]. A 567 Mbp reference sequence [18] with 26,923 annotated protein-coding genes [19] has recently been published. The taxonomic position of *B. vulgaris* lies within the Amaranthaceae family which belongs to the order Caryophyllales. In contrast to species from the asterid and rosid clades, the red, violet and yellow pigments in species of the *Beta* family are derived from the betalain biosynthetic pathway (see [20] and references therein). The capability to produce betalains is unique to some families of the order Caryophylalles [21]. Betalain accumulation in *B. vulgaris* is controlled by at least two loci, called *R* and *Y*, which are under investigation since the 1930s [22]. *R* was found to control the red versus yellow shift in beets, whereas the *Y* locus is responsible for pigment versus no pigment in the interior of the beet root [23]. *Y* was found to be separated from the *R* locus by a genetic distance of 7 cM [22]. The two loci were identified and molecularly described in 2012 [24] and 2015 [25], respectively. Usually, the *R* locus contains a dominant allele that conditions a red hypocotyl. Since *R* is assumed to be unlinked to other regions that are under selection during sugar beet breeding, the *R* locus has also been studied because of its role as a visible marker in crosses. The gene *BvCYP76AD1* that represents the *R* locus was referred to as the *RED* gene. It codes for a cytochrome P450 enzyme that is required for betalain biosynthesis [24]. The characterized mutant allele *r* contains a 5 bp long insertion in the coding region of the gene leading to a premature stop codon. This loss of function mutation in the recessive allele *r* results in a lack of betalain accumulation in the hypocotyl, causing a green to yellowish hypocotyl phenotype that is easy to score (Fig. 1).

Using the hypocotyl color trait, we show that MBS can be applied to phenotypic pools from breeding panels to successfully identify a narrow genetic interval that contains a causal gene in the crop *B. vulgaris*. Since the plant lines displaying the contrasting red or green hypocotyl color phenotype were selected from breeding panels, multiple rounds of crossing, recombination and selection were accessed in a convenient way. By selection of homozygous inbred or DH lines we avoided the need to deal with plants that are genotypically heterozygous *red*/*RED*. The identified genetic interval had a size of 31 kbp and contained the known *RED* gene encoding *BvCYP76AD1*.

## Results and discussion

The starting point for the experiment to map the *R* locus of *B. vulgaris* by MBS was the selection of the plant material. A total of 360 different homozygous accessions that had been phenotypically characterized for hypocotyl color (Fig. 1) were selected. Of these, 180 displayed green hypocotyl and were expected to be genetically *rr*, while the other 180 accessions displayed red hypocotyl and were expected to be genetically *RR*. The accessions were randomly selected from the breeding programs of three sugar beet breeding companies each contributing 60 *rr* (green) and 60 *RR* (red) accessions. Since it was initially unknown if all three panels really contained the same *r* allele (i.e. the identical mutation at the *R* locus), DNA was extracted for each of the six pools of 60 accessions separately. Genomic DNA of each of the six pools was sequenced with Illumina PE technology (see Methods) with the goal to reach a target coverage of about 25 fold, resulting in an overall coverage of about 75 fold for both superpools that were created *in silico*.

**Fig. 2:**
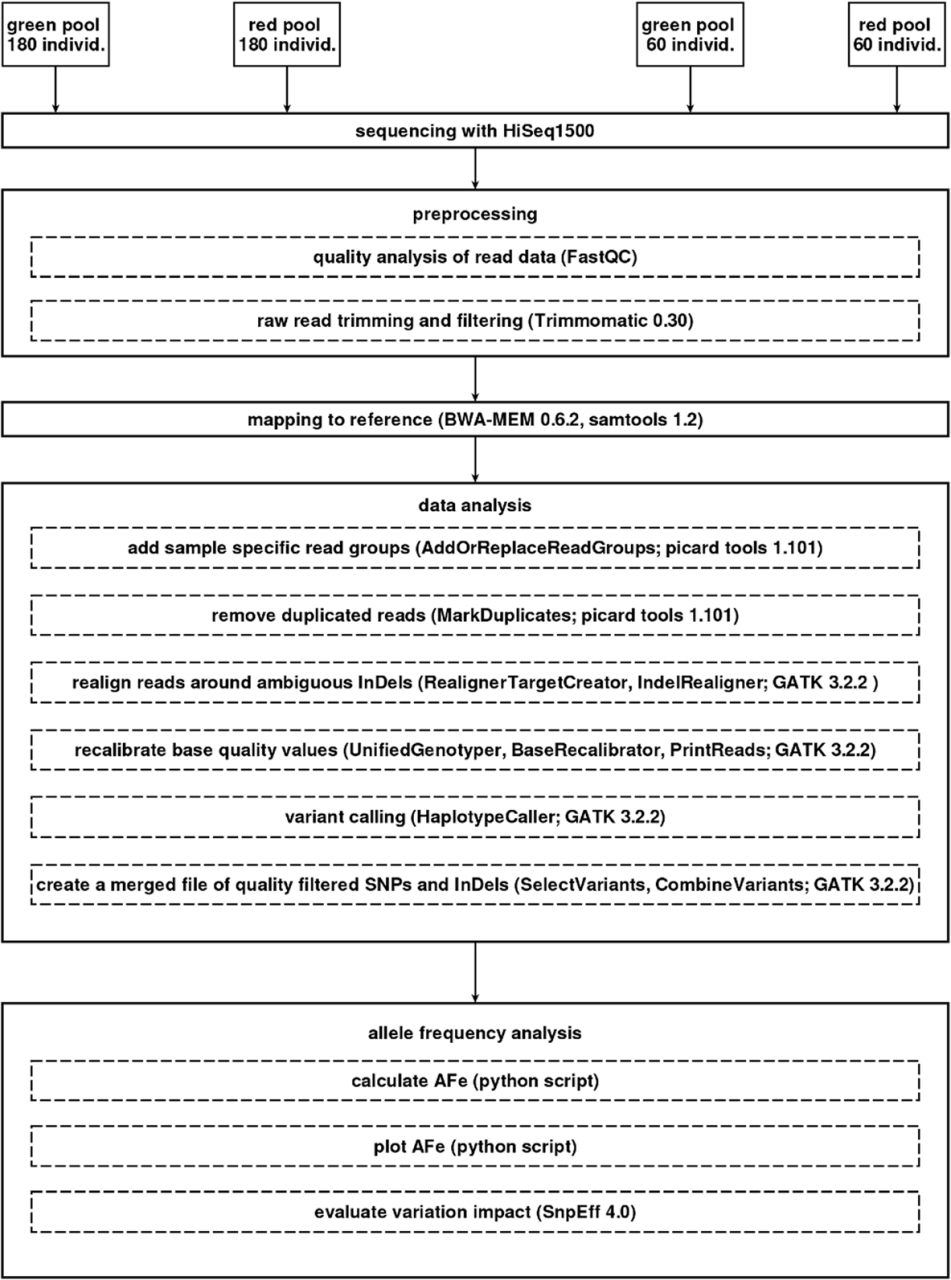
Experimental setup and work-flow. Overview of the bioinformatic processing steps, starting with sequence data from different DNA pools. Program or tool versions used are indicated. For a more detailed description of the single steps, see Methods

After stringent quality filtering the coverage of uniquely mapped reads was close to the planned values (Table 1). For the visualization and detection of a local skew in the allele frequency, it was crucial to detect the changes in allele frequency of sequence variants. Therefore, the read data were mapped to the sequence of pseudo-chromosomes which we derived from the ordered scaffolds of the reference sequence [18]. All steps of the data analysis pipeline, from read mapping to variant calling, were implemented to run on a local compute cluster (see Methods). Figure 2 depicts the work-flow from sequencing of the pools via data processing to evaluation of variants. Due to the sampling of accessions into pools and inherent properties of short-read sequencing methods, like stochasticity in sequencing coverage or random sampling effects for read coverage at a given polymorphic position, the values of the true allele frequencies can only be estimated [26]. We used the term allele frequency estimate (AFe) to reflect this fact (see Methods for the definition of the term AFe and delta-AFe). The final set of identified high quality biallelic variations consisted of 5,470,336 SNPs and 964,904 InDels. The results of the individual steps to the final set of variations are described in detail below.

**Table 1:** Illumina read data used and coverage of the concatenated RefBeet1.2 reference sequence

	Depth of coverage ^a^	Read length ^b^	Insert size ^c^	Reference covered ^d^
Breeding panel 1 red	24.72 x	150	500	66.5
Breeding panel 1 green	23.60 x	150	500	62.0
Breeding panel 2 red	25.60 x	150	500	64.4
Breeding panel 2 green	25.08 x	150	500	63.0
Breeding panel 3 red	19.41 x	150	500	47.2
Breeding panel 3 green	23.45 x	150	500	62.9
Breeding panel 2 red total	73.27 x	100/150	450/500	90.0
Breeding panel 2 green total	69.69 x	100/150	450/500	89.3
Superpool red	76.14 x	100/150	450/500	91.7
Superpool green	78.41 x	100/150	450/500	91.9

^a^ Mean number of uniquely mapped reads covering each base of the reference sequence

^b^ Length of the sequenced reads in base pairs; if more than one number is shown, two datasets with differing read length were merged; all generated as paired ends (PE)

^c^ Targeted length of the PE sequenced fragments in base pairs; if more than one number is shown, two datasets with differing read length were merged

^d^ Percentage of the reference sequence with more than 15 fold read coverage

Theoretically, it is possible that the mutated nucleotide position(s) which cause the *r* allele to be non-functional are also affected in functionally intact *R* alleles. This would, since multiallelic nucleotide variations for a single SNP or InDel were excluded, result in removal of the causal variant from the data. However, we considered this risk very low given the genetic background of the material, and the fact that independent mutations affecting identical positions are very rare. We determined the rate of positions with more than two alleles in our data to be about 0.1 % for SNPs. In most of the cases, these multiallelic nucleotide variations might be caused by errors in variant detection.

### Improvement of InDel detection

Obviously, not only the relatively easily detected SNPs but also InDels have the potential to be causal for a given trait. In addition, InDels are also useful for determination of additional AFe values and contribute to an increased marker density. Therefore, a correct mapping of reads to regions containing InDels was crucial. Unfortunately, unprejudiced read mappers including BWA can only map reads correctly that cover both sides, upstream and downstream, of a given InDel. Reads that either begin or end close to an InDel are often mapped incorrectly and can, therefore, contribute wrongly to either of the true allelic options that exist at the studied position. Often, the real insertion or deletion thus appears to be less abundant than it truly is. The bioinformatics toolbox GATK [27] offers the tool IndelRealign that locally re-aligns reads at InDel positions, so that the number of mismatching bases is minimized across all reads spanning a given InDel. This tool removes many misalignment artifacts, resulting in significantly improved AFe values for InDels and also for SNPs in their immediate vicinity. Correct determination of AFe values is especially relevant for the detection of the causative InDel or SNP, because this should, in theory, display an AFe of close to the value 1. Since the mutation at the *R* locus had previously been identified as an InDel in a somehow repetitive sequence [24] that is even more affected by mismapping, application of the local read realignment tool was obligatory for success of MBS in our case.

### Improvement of variant calling and variant filtering

To further improve the accuracy of variant calling, GATK’s Base Quality Score Recalibration (BQSR) was applied. BQSR requires a training set that was unavailable for *B. vulgaris*, and consequently a training set was created. To account for run-specific errors, and because variants may occur only in one pool and with low frequency, a separate training set for each sequencing run was created. This should avoid treatment of diluted variants as errors after merging of the data. After recalibration of the quality scores by BQRS, the sequencing runs were merged into one set of reads. The final variant calling was done with HaplotypeCaller (see Methods).

Variant calling identified 8,202,758 variations (7,006,049 SNPs and 1,201,615 InDels). After filtering according to the “GATK best practice” (see Methods) to reduce false positive variant calls, as well as exclusion of multiallelic nucleotide variations, 5,471,088 SNPs and 965,211 InDels were considered as high confidence variations between the combined sequence data from all breeding panels and the *B. vulgaris* reference sequence. The removal of multiallelic nucleotide variations affected 24,977 polymorphic positions. For the final comparison of the pools, we could only consider variations for which we had sequence information from both pools. After filtering out variations that were only covered in one of the two superpools, a final set of 5,470,336 SNPs and 964,904 InDels was obtained.

### Identification of a 31 kbp interval containing the RED gene

**Fig. 3:**
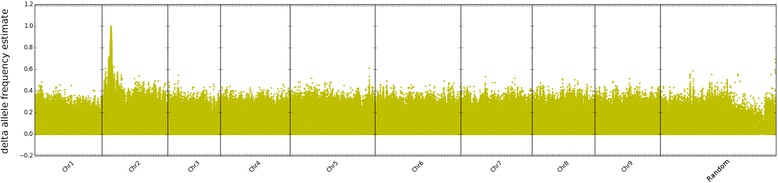
Allele frequency estimate (AFe) of the red versus the green pool of 180 accessions each plotted as delta-AFe values. The delta-AFe values for all detected variants (yellow dots) were plotted along the concatenated reference sequence sorted according to chromosome number. *B. vulgaris* chromosome 2 shows a clear difference peak close to it’s upper end (according to the standard orientation). Since the calculated AFe values become less reliable with less coverage, only variants supported by a mapped coverage of between 0.75 and 2.5 fold of the mean coverage of the respective chromosome were included in the plot

**Fig. 4:**
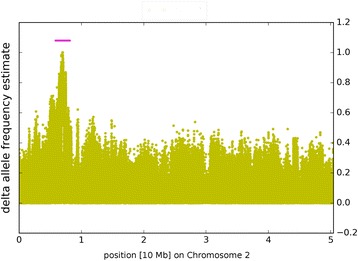
Plot of delta-AFe values for chromosome 2. The delta-AFe values, indicated by yellow dots, clearly show a skew in the AFe values of the two pools. The pink line indicates the ad-hoc identified interval surrounding the peak

For easy visual identification of genetic intervals showing deviant AF introduced by the phenotypic selection when building the pools, the AFe values of all detected variants were plotted along their position on the nine sugar beet pseudo-chromosomes. Inspection of the sequence data had shown that all three breeding panels contained reads derived from the published *r* allele [24]. Therefore, data from the three breeding panels were combined, and AFe values from the ‘red’ and the ‘green’ superpool were analyzed. To emphasize that the most relevant information to be extracted from phenotypic pooling is the separation of alleles into the two pools, delta-AFe values were determined. The mean delta-AFe is around 0.08 for each of the nine chromosomes, except for chromosome 2, for which it is 0.12. Figure 3 shows the result of plotting the absolute values of delta-AFe along the genome sequence, represented by concatenated pseudo-chromosomes. Inspection of the plot allowed ad-hoc identification of a clear peak of the delta-AFe value distribution at the top of chromosome 2. The result of plotting the delta-AFe values for an expanded pseudo-chromosome 2 only is shown in Fig. 4.

A list of all variants with an AFe value close to zero in the ‘green’ superpool (see Methods for a description of the selection of seed variants and the algorithm for interval definition) was extracted from all AFe data. These variant positions were used as starting points for interval detection. This resulted in detection of a single interval on chromosome 2 with an exact length of 31,435 bp, overlapping the central part of the peak visualized by plotting delta-AFe values. Non of the other chromosomes displayed any variants with AFe values close to zero in the “green” superpool, so no further intervals were defined.

**Fig. 5:**
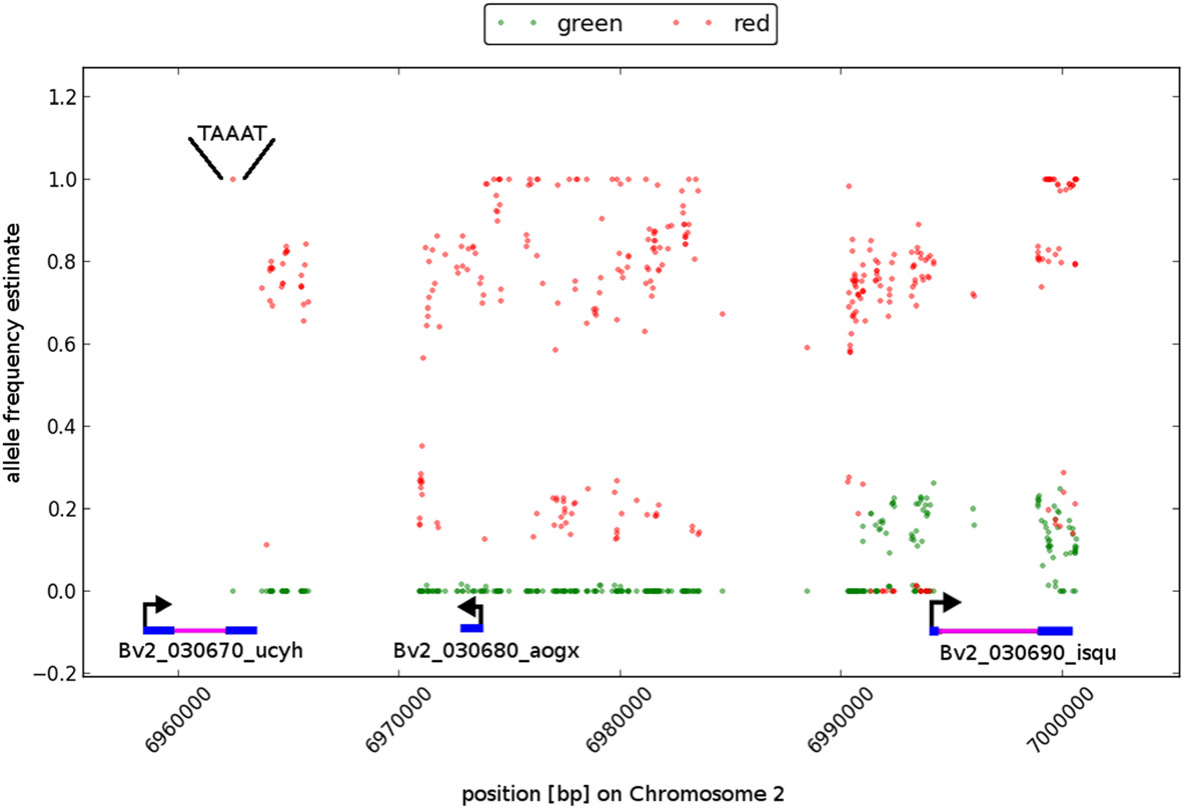
Plot of AFe and delta-AFe values for the genetic interval detected. AFe values of the variants detected in the identified interval were plotted for the ‘red’ and the ‘green’ superpool, with a red dot on top of a green one if both were at an identical spot. The intron/exon structure of genes (exons blue, intron pink) overlapping the interval are depicted at the bottom of the figure. The InDel bases present in the sequences of the 'green' pool and absent from the sequences of the 'red' pool are shown in the upper left area. This InDel affects the gene Bv2_030670_ucyh which is the *RED* gene. Note that the accession KWS2320 from which the reference sequence has been derived was green and genotypically *rr*

With a focus on the interval detected, AFe values from the ‘red’ and the ‘green’ superpools were plotted (Fig. 5). The distribution shows that variant positions in the interval from the ‘green’ superpool follow the reference sequence, while the variant positions from the ‘red’ superpool show fluctuating AFe values. This fits to the expectation because the DH genotype KWS2320 that provided the reference sequence is genetically *rr* and phenotypically green. The gaps in the variant distribution, i.e. the regions with no AFe values plotted in Fig. 5, were probably mostly caused by the unique read mapping requirement applied during variant detection, causing no mapping coverage in repetitive, paralogous or duplicated regions.

### Identification of the causal gene from three genes in the interval

The interval on chromosome 2 of 31,435 bp in length overlaps three annotated genes, of which one is the published *RED* gene *Bv2_030670_ucyh*. As expected, all AFe values from the green superpool were close to zero since this criterion was used for interval detection. However, most AFe values from the ‘red’ superpool were ranging between 0.3 and 1. Thus, the data demonstrated the existence of exactly one ‘green’ haplophase (the *r* allele) identical to the reference sequence, but multiple ‘red’ phases or *R* alleles that contribute individual combinations of variant positions. Of all variants within the interval, only one had a delta-AFe of 1 *and* was overlapping an exon. This single variant was identical to the causative 5 bp insertion in the gene *Bv2_030670_ucyh*. The insertion was present in all reads from the ‘green’ superpool mapping to the respective variant position, and in the reference sequence resulting in the determination of an AFe value of 0 (zero). At the same time, the causative 5 bp insertion showed an AFe of value 1 in the ‘red’ superpool. These results are in full concordance with the previous identification of the *RED* gene [24].

The two other genes, *Bv2_030680_aogx* and *Bv2_030690_isqu* that are also contained within the defined genetic interval would also be candidates if the causal gene would not have been identified already. However, they were less probable candidates compared to *Bv2_030670_ucyh* for the following reasons. First, the CDS regions of both of them do not carry variant positions that display delta-AFe values close to 1 (one). Second, *Bv2_030690_isqu,* which is located at the downstream border of the interval, carries quite some variant positions in its 5′ region that display clearly AFe values significantly higher than the expected value of zero. Manual inspection of the reads supporting these values of about 0.2 confirms that these AFe’s are real. Third, *Bv2_030680_aogx*, although in the middle of the interval, does not overlap with any strongly segregating variations (the ‘red’ AFe values within the gene were all significantly below 1). There were some variants outside annotated genes (e.g. in the area around position 6,980,000) that show delta-AFe values of 1, but based on the assumption that the green hypocotyl phenotype is caused by a gene affecting pigmentation these positions can be considered less relevant. Finally, we analyzed and categorized the effect of sequence variations on the coding regions of the three genes within the interval with SnpEff [28]. The gene *Bv2_030670_ucyh* was indeed the only gene affected by a variant that most likely has a deleterious effect, indicating that even without knowledge about the causal mutation the gene *Bv2_030670_ucyh* would have been identified as the best candidate.

### The number of accessions used has a high influence on mapping resolution

**Fig. 6:**
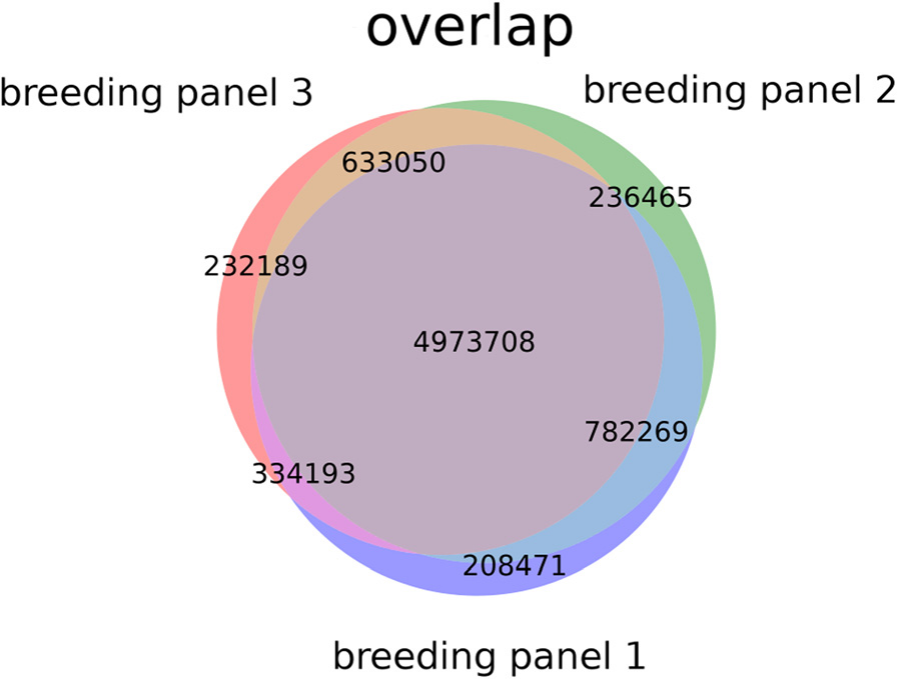
Overview of common and independent variations in the breeding panels studied. Venn diagram of shared and private variations detected by pool sequencing. Only variations for which information (read coverage) was available in all three pools independently were taken into account

To determine the effect of using either 120 (two times 60) or 360 (two times 180) accessions, the differences in AFe values from the two superpools generated with 180 individuals were compared to those from one of the pools generated from only two times 60 individuals. To select one of the three breeding panels for this experiment, the mean AFe value for the combined (‘green’ plus ‘red’) pools from the three panels was determined (see Methods) with the goal to identify the panel with the material that was the least similar to the reference sequence. The two ‘red’ and ‘green’ superpools showed a mean AFe value of 0.37. Breeding panel 2 displayed a mean AFe value of 0.44, while the two other panels showed mean values of 0.35 and 0.37, respectively. Breeding panel 2 also showed the highest number of variants exclusive to one breeding panel, and the smallest overlap with the other breeding panels (Fig. 6). During this evaluation, we also noted that more than 80 % of the same biallelic variant positions were detected in all breeding panels.

**Fig. 7:**
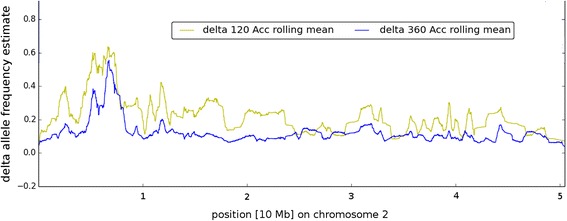
Delta-AFe plot to compare results from pools of 360 vs. 120 accessions. Sliding windows (2.5 kb window size, 0.5 step size) of delta AFe values of detected variants were plotted along chromosome 2 from the two different pools studied. Colors as indicated

The two pools (‘green’ and ‘red’) from breeding panel 2 which diverged with a mean AFe value of 0.44 were chosen for further sequencing to reach a coverage comparable to that of the two superpools (about 70 fold uniquely mapped coverage). Variants were called for both pools with 60 accessions, and delta-AFe values were determined. For comparison to the results from the superpool analyses that was based on two times 180 accessions, a sliding window approach was used (see Methods). The results are summarized in Fig. 7. The data showed that using 60 accessions per pool was still sufficient to identify the correct genomic region. However, the interval detection procedure also applied to the superpool data now yielded four intervals (length in bp: 33,930; 28,736; 20,920; 14,474 with gaps in between) that were distributed over almost 360 kbp. In total, the genetic interval detected with 2 × 60 accessions was about 14 times larger than the interval from full data. Also, the reduction of the number of accessions from other breeding panels has strongly increased background noise, also contributing to a much broader peak in the target region. The sharper peak and shorter interval with 2 × 180 accessions were a result of the higher number of recombinations introduced with more accessions.

Attention should be paid to the fact that it was not necessary to generate a read coverage high enough to capture all individuals in the pools, but only high enough to get a good representation of the distribution of frequent variants within the respective pool. A coverage of about 70 fold was sufficient to capture information from 180 accessions (coverage about 0.4 per accession). Since 70-fold coverage is by far not enough to sequence every chromosome in the pool, rare variants that occur in only a few of the accessions are underrepresented in our data set. By increasing the number of accessions these rare variants, that are “private” to a single or a very low number of accessions, get further suppressed. Under these conditions, such rare variants were not distinguishable from sequencing errors. This also contributes to a reduction of the variance of AFe, lowering what looks like background noise in our approach, and exposing the interval of interest. In other words, the coverage we used introduced an intended bias against rare variants. Due to the accumulation of historic recombinations in each accession, the effect of adding more accessions to the pools is stronger than adding more F2 individuals from a crossing experiment. Thus, our approach yielded a really short interval, probably much shorter than from a MBS approach using a similar number of F2 individuals.

### About 30 fold coverage is sufficient for interval detection

To test the effect of increased coverage on interval detection while keeping the number of accession at 2 × 180, we applied our method to subsets of the data. Nine subsets of the mapped reads of the two superpools were generated, with linearly increasing coverage and an increment of 1/10 of the total coverage (Table 2). The resulting mapping data were used to call variations. Putative intervals were then defined by the algorithm described above.

**Table 2:** Intervals detected for subsets of the superpools with increasing coverage

Coverage ^a^	# intervals ^b^	Length of intervals ^c^	Filtered intervals ^d^
~7.8	7	145391	107
~15.5	2	59660	77
~23.3	1	48369	40
~31.1	1	29162	19
~38.9	1	29162	9
~46.6	1	29162	4
~54.4	1	29162	0
~62.2	1	29162	0
~69.9	1	29162	0
~77.0	1	29162	0

^a^ Mean number of uniquely mapped reads covering each base of the reference sequence

^b^ Number of intervals identified

^c^ Summarized length of identified intervals in base pairs

^d^ Number of discarded intervals

With a coverage of about 23 fold, the correct interval was found, but this interval was about 25 % larger than the one identified with the full coverage. The correct interval with the correct length was identified starting at an average coverage of about 31 fold. However, at this coverage 19 additional “seeds” were detected but not extended to putative intervals because the regions around them stayed shorter than 500 bp (see column “filtered intervals” in Table 2). Using a coverage of more than about 54 fold did not yield further improvements of interval detection. A similar lower limit of mapped read coverage of 30 fold for variant detection has also been concluded from a study that compared various SNP and InDel detection experiments [29].

Another difference between the mapping results from the different subsets was the portion of the genome covered adequately with reads. As can be seen in Table 1, a coverage of 70 fold was sufficient to cover over 90 % of the genome with at least 15 reads, which we consider the minimum for reliable variant calling and AFe value determination. Due to stochastic effects and sequencing biases in certain regions of the genome, the proportion of the genome sufficiently covered with mapped reads is reduced with lower overall coverage. In a MBS experiment where two pools have to be compared, reliable sequencing data has to be available in both pools for each position to compare. This can lead to significant data loss, if a random distribution of reads is assumed. For example: if the fraction of adequately covered sequence in each pool is 60 %, the fraction of the genome available for reliable comparison of AFe values and interval detection might be reduced to only 36 % (0.6 × 0.6 = 0,36 as the theoretical extreme). Therefore, it is important to perform initial calling of variant positions for the combined pools, so that the reads from both pools support each other for variant detection. With this approach, which is implemented in the GATK toolkit, the coverage can virtually be combined, and high quality variations for both pools can be called for a larger portion than if the pools were analyzed individually. Still, the proportion of the genome covered by reads needs to be considered when planning a MBS experiment and the targeted coverage.

## Conclusions

The NGS technology has improved forward genetics to an extent that has made the identification of causative mutations of a phenotype fast and straightforward. Although there are probably still problematic cases, success has been shown numerous times [9–13, 30]. In most of these cases, the schedule applied did require sequencing of parental lines, generation of F2 or F3 individuals, backcrossing of mutant lines, prior knowledge of the location of the causative mutation or the use of existing markers. We carried out deep-sequencing of two phenotypic pools of 180 sugar beet accessions each from three breeding panels, and performed genome-wide analyses of differential AFe values between the two pools. In our proof-of-principle case, a very small genetic interval of 31 kbp containing the causative gene [24] for the green hypocotyl mutant phenotype was identified, and careful examination of the results indicated that additional evidence like categorization of SNP/InDel effects might have allowed to predict the correct gene even if it would have been unknown.

Our approach allowed to identify a causative locus for a phenotype, in general within a few weeks after plant samples are harvested. Neither prior knowledge nor additional sequencing of single offspring genotypes or parental lines was necessary. Relatively high depth sequencing of the pools to at least 30 fold coverage was needed to get a reliable signal, and an increase of the coverage to 50 or 70 fold reduces the risk that the relevant genome region is less well covered by chance. Extensive post processing of sequence data, read mapping, variation calling and exact determination of AFe values, especially for InDels, was essential for success. The GATK toolkit [27, 31] turned out to be a flexible and well adaptable tool to perform these processing steps that we successfully applied for a crop plant genome.

Obviously, the approach relies on the availability of appropriate material, in our case phenotyped accessions of diverse genetic origin that share one mutant allele. For easy identification of the causal mutation the homozygous state of the plants in the pools was necessary. However, modern plant breeding often uses lines that have been bred to full homozygosity or applies double-haploid (DH) technology so that useful genotypes for various phenotypes might be available in breeding panels. The next step would be to determine how heterozygosity in the pool derived from the dominant phenotype will affect the identification and resolution of the genetic confidence interval for other traits of interest in crop plants.

## Methods

### Selection of plant material

A total of 360 individual accessions were selected, with either red or green hypocotyl color, respectively. All accessions were from the species sugar beet (*Beta vulgaris* subsp. *vulgaris* var. *altissima*), and all were either DH or inbred lines. The accessions were phenotyped for the hypocotyl color trait known to be located on chromosome 2. Both phenotypic groups of 180 accessions each were derived from three separate breeding panels (referred to as breeding panels 1, 2 and 3), with each of the breeding panels providing 60 ‘red’ and 60 ‘green’ individuals, in total 120 accessions from each breeding panel.

### Plant growth and DNA isolation

Plants were grown in the greenhouse under long day conditions on soil. Each accession was represented by a single plant. Equal amounts of leave tissue per plant were collected from the 3rd and 4th real leaf. Two leave disks per plant were sliced out by applying a cork driller with a diameter of 1 cm. The material from accessions with the same phenotype and from one breeding panel was combined at harvest. Genomic DNA from six pools (the ‘red’ and ‘green’ pools from breeding panel 1–3) was isolated with the Epicenter gDNA Isolation kit according to the manufacturers instructions. Subsequently, the DNA obtained was treated with RNAse and quantified using PicoGreen.

### Library preparation and sequencing

Library preparation for all DNA pools was performed according to the Illumina TruSeq DNA Sample Preparation v2 Guide. DNA from each pool was fragmented by nebulization. After end repair and A-tailing, individual indexed paired end (PE) adapters were ligated to the DNA fragments which allowed multiplexed PE sequencing. The adapter-ligated fragments were size selected on a 2 % low melt agarose gel to a size of 500–800 bp. After enrichment PCR of fragments that carry adapters on both ends the final libraries were quantified by PicoGreen. The average fragment size of each library was determined on a BioAnalyzer High Sensitivity DNA chip. Samples from each of the six libraries were pooled in equimolar amounts and sequenced on a HiSeq1500 in rapid mode as well as in high output mode. Clusters on the flowcell for rapid runs were generated by on-board cluster generation using the TruSeq Rapid PE Cluster kit and sequenced according to the 2 × 151 PE scheme using TruSeq Rapid SBS chemistry. Cluster generation for high output runs was carried out on a cBot using the TruSeq PE Cluster kit v3, and sequenced according to the 2 × 101 scheme using the TruSeq SBS Kit v3. After completion of the sequencing runs, basecalling, demultiplexing and fastq file generation was performed using a CASAVA-based inhouse script.

### Generation of a reference sequence with pseudo-chromosomes

For mapping of the sequencing reads, a modified version of the published RefBeet1.2 [GenBank: AYZS00000000.1] reference sequence [18] was generated. The scaffolds of each chromosome of the original RefBeet-1.2 reference sequence were concatenated, and stretches of 50 N’s were inserted between scaffolds to form pseudo-chromosomes. Scaffolds that are assigned to a chromosome, but not anchored to a fixed position on the given chromosome, were appended in the same way, resulting in a ‘random’ pseudo-chromosome part at each downstream (bottom according to standard orientation) end of the chromosomes. Unassigned scaffolds and contigs were concatenated in the same way, resulting in a pseudo-chromosome designated ‘Random’. The modified sequence was named RefBeet-1.2-joined. The python script “concat_contigs.py” which was used to generate the sequence from the RefBeet1.2 is available as Additional file 1, the zipped archive also contains a ReadMe (concat_contigs.README.txt) which contains some instructions as well as a parameter file.

### Postprocessing and mapping of reads from pools

All operations from postprocessing to variant calling were performed on the compute cluster of the Bioinformatics Resource Facility of the CeBiTec. GATK allows for distributed data processing by default [27]. Parallelization by using multiple cores or distribution to multiple nodes was applied when ever possible, allowing the full compute process to be finished within less than 1 week. An overview of the workflow is presented in Fig. 2.

For adapter trimming, quality filtering of reads with stretches of four consecutive bases with a mean quality value below 30, and removal of bases at the read heads and tails with quality values below 25, the tool Trimmomatic [32] was applied. After trimming, the data was quality checked using FastQC [33]. The trimmed reads were mapped to RefBeet1.2-concat with BWA, using the MEM algorithm [34] that is adapted for longer reads. Default parameters were applied. To improve mapping speed, the data were split into chunks that were processed separately on the compute cluster using a divide and conquer approach. The mapping results were merged into one file of the .bam format [35] containing all reads. Finally, mapped reads were filtered for duplicates with PicardTools [36].

Due to variation of sequencing yield, the coverage of mapped reads was initially about 20 fold for the ‘red’ and the ‘green’ pool from breeding panel 1 and 3, and about 30 fold for the pools from breeding panel 2. For comparison of results when different numbers of accessions were used, additional sequencing was performed for both pools from breeding panel 2 to reach an overall coverage of mapped reads of about 70 fold.

### InDel realignment

Since unprejudiced read mapping tools cannot always decide how to correctly map a read that begins or ends in an InDel, realignment of reads at such positions increases the confidence of subsequent processing steps. The GATK InDel realignment tool [31] from the GATK toolbox [27, 37] adjusts the aligned reads at ambiguous sites in a way that the number of mismatching bases of all reads is minimized. We applied GATKs RealignerTargetCreator to identify suspicious intervals and then applied the IndelRealigner to those intervals, using GATKs standard parameters.

### Base Quality Score Recalibration

The Base Quality Score Recalibration (BQSR) tool of the GATK optimizes quality scores for the reads in a .bam file. Quality values are recalibrated to fit closer to the real probability of mismatching the reference. In addition, effects of machine cycle and sequence context are taken into account during recalibration. To separate mismatches into variations and sequencing errors, the base quality recalibrator needs a file of real, verified variations. Such a file was not available for sugar beet. Following GATK’s best practice recommendations, we created a file of high confidence polymorphic sites by calling variations with GATK’s UnifiedGenotyper and hard filtering the results. Parameters for filtering were selected according to GATK’s best practice workflow. The newly generated list of high confidence variants was subsequently used to recalibrate the quality values of the mapped reads with BQSR.

### Variation calling

Data were processed with standard tools including SAMtools [35] for format conversion, sorting and indexing and PicardTools for manipulation of read groups and assessing data metrics. The mapped and filtered reads were further processed using the GATK pipeline. We found that GATK produced highly reliable variant calls, at least for our dataset. The complete best practice workflow of GATK [27] was applied. Correct AFe (Allele Frequency estimate) values for InDels were only obtained after application of GATK’s HaplotypeCaller. Variations were called for both pools together with GATK’s standard parameters for hard filtering. Only reads with a mapping quality higher than 0 were used for variant calling, thus excluding non-uniquely mapped reads. Multiallelic variants were excluded. Finally, two files were obtained containing high confidence, biallelic SNPs and InDels, respectively. SNPs and InDels were merged into one file and only variant positions that were covered in both pools were considered for further analysis.

### Calculation of allele frequencies

AFe values were calculated from the relative amount of sequence reads supporting either of the two alternative alleles for any given variant position. An AFe of 0.9 results if 90 % of all reads covering the addressed variant position support the alternative allele (on the basis of the KWS2320 reference sequence), and 10 % the reference allele. Since the sampling of the reads follows an almost random distribution (values cannot be smaller than 0 or larger than 1), the AFe value is an approximation of the real allele frequency at the studied position. The fit of AFe values to the real allele frequency, or to the true proportion of chromosomes in a pool carrying two alternative alleles at a given position, becomes more precise with increasing read coverage. Allele frequencies should become increasingly more divergent between the two phenotypically selected pools for positions that are closer to the causative locus for the phenotype addressed. At the causative locus itself, the maximal difference of 1 for a variant with two alleles of which each is “private” to one of the two pools. To emphasize this, the delta allele frequency estimate (delta-AFe) was determined. Delta-AFe was calculated as the absolute difference between the AFe values from the two pools for a given variant position.

### Generation of an error file for the reference genotype

During data analyses it turned out that some high AFe values that were detectable in both superpools were caused by potentially erroneous bases contained in the sugar beet reference sequence of the genotype KWS2320. To lower the number of these false positive data points, we generated a list of these potentially erroneous (or at least different) base positions in the reference sequence and removed these positions from the final list of variations called for the MBS experiment. The reference sequence is based on a backbone of sequence data generated by 454 technology [18] but the MBS data in this study were generated with Illumina technology. We used Illumina sequence reads from KWS2320, called variations using the CLC genomics workbench which yielded about 25,000 potentially questionable positions, and used these results to prepare the list mentioned above.

### Interval identification and prediction of causal variations

For visual inspection of the results, the AFe values of detected variants were plotted along all chromosomes or for selected regions, usually for both pools into one plot. To reduce background noise, only variants with a coverage between 0.75 and 2.5 times the average coverage of uniquely mapped reads were plotted. The reasoning for these limits was that lower coverage contributes to higher error in the deduced AFe values due to stochastic effects, and significantly higher than expected coverage at a given position indicates that the respective region in the reference sequence might contain collapsed repetitive sequence. To allow easy identification of positions with differing AFe values, the difference of the AFe values (delta-AFe) between the two corresponding pools was plotted for each variant position, using the python script “VCF2AFAnalysis.py”, which can be found in Additional file 2. The output allows ad-hoc delineation of relevant intervals. However, an algorithm for automatic identification has also been implemented.

An interval is defined as a genome region containing a series of variants with AFe values of 0.1 or lower in the ‘green’ pool. Interruptions of the series are ignored if they consist of at most 1 variation with an AFe larger than 0.1, which is flanked on each side by a variant with AFe values of 0.1 or lower. Interval detection was started at seed variants with an AFe value close to zero in the green superpool. The exact, lowest delta-AFe value of valid seeds (X) was calculated depending on sequencing error rate and coverage to tolerate a small number of non-supportive reads. *X* = (P - 100 * E/C) with phenotypic difference of the pools *P* = 1, the coverage of both pools combined for the variant C, and an estimate for sequencing errors introduced by the HiSeq1500 *E* = 0.01. Intervals shorter than 10 kbp were discarded. We filtered all variants within the detected intervals for GenotypeQuality (GQ) values better than 20, and for displaying a delta-AFe of more than 0.9, thus being homozygous differences between the two pools. SNPeff [28] was used to predict the effect of those variants on genes overlapping the interval.

### Calculation of mean allele frequencies for single breeding panel pools

To calculate the mean allele frequencies for the pools of each breeding panel separately, we extracted the variants called for each panel from the variant file deduced from the superpools. For this, we used GATK’s SelectVariants tool, and filtered for homozygous and heterozygous variants of one breeding panel respectively. The selected variants were then coverage filtered as described above (coverage between 0.75 and 2.5 times the mean coverage of the respective chromosome). Finally, the mean genome wide allele frequency was calculated.

### Comparison of mapping resolution from 360 versus 120 accessions

The variations of the breeding panel specific pools of 120 (2 × 60) accessions were filtered with the same parameters as described for the superpools of 360 (2 × 180) accessions. We applied a sliding window with 2.5 kb window size and 0.5 kb step size to the delta-AFe values of both experiments. The results were then plotted together along the pseudo-chromosomes. For coverage filtering, calculation of delta-AFe values, application of the sliding window and plotting of the data, we used the python script described above.

### Generation and comparison of reduced coverage data subsets

Randomized subsets of reads from the superpools .bam file were generated using GATKs PrintReads with the -dfrac option. The targeted partial coverage was increased stepwise with an increment of 1/10 of the total coverage, starting with about 8 fold coverage (see Table 2). The resulting .bam files were submitted to our analyses pipeline starting with variant calling. The final, filtered variations were used for interval detection by applying the algorithm describe above.

## Availability of supporting data

The data set supporting the results of this article is available from the European Variation Archive (EVA), with the accession number PRJEB11641 (URL: http://www.ebi.ac.uk/eva/?eva-study=PRJEB11641).

## Additional files

Additional file 1: “concat_contigs.py” script for generation of RefBeet-1.2.joined.fa. The python script “concat_contigs.py” and supporting files were used to generate the concatenated sequence RefBeet-1.2.joined.fa from RefBeet-1.2.fna [GenBank:AYZS00000000.1] [18]. It was tested on Unix/Linux systems with at least python version 2.7 installed. (ZIP 2 kb) Additional file 2: Python package to generate AFe plots and automatic identification of intervals. The python script “VCF2AFAnalysis.py” was used to generate AFe plots from vcf files. Usage examples can be found in the “README” text file contained in the package. (ZIP 18 kb)

## Abbreviations

AFe: Allele frequency estimate
BQRS: base quality recalibration step
BSA: bulk segregant analysis
InDel: insertion deletion
MBS: mapping by sequencing
NGS: next generation sequencing
SNP: single nucleotide polymorphism
VQSR: variant quality score recalibration

## References

[1] Waugh R, Leader DJ, McCallum N, Caldwell D (2006). Harvesting the potential of induced biological diversity. Trends Plant Sci.

[2] Michelmore RW, Paran I, Kesseli RV (1991). Identification of markers linked to disease-resistance genes by bulked segregant analysis: a rapid method to detect markers in specific genomic regions by using segregating populations. Proc Natl Acad Sci U S A.

[3] Becker A, Chao DY, Zhang X, Salt DE, Baxter I (2011). Bulk segregant analysis using single nucleotide polymorphism microarrays. PLoS ONE.

[4] Quarrie SA, Lazic-Jancic V, Kovacevic D, Steed A, Pekic S (1999). Bulk segregant analysis with molecular markers and its use for improving drought resistance in maize. J Exp Bot.

[5] Sham P, Bader JS, Craig I, O'Donovan M, Owen M (2002). DNA Pooling: a tool for large-scale association studies. Nat Rev Genet.

[6] Varshney RK, Nayak SN, May GD, Jackson SA (2009). Next-generation sequencing technologies and their implications for crop genetics and breeding. Trends Biotechnol.

[7] Bolger ME, Weisshaar B, Scholz U, Stein N, Usadel B, Mayer KF (2014). Plant genome sequencing - applications for crop improvement. Curr Opin Biotechnol.

[8] Schneeberger K, Weigel D (2011). Fast-forward genetics enabled by new sequencing technologies. Trends Plant Sci.

[9] Schneeberger K, Ossowski S, Lanz C, Juul T, Petersen AH, Nielsen KL, Jorgensen JE, Weigel D, Andersen SU (2009). SHOREmap: simultaneous mapping and mutation identification by deep sequencing. Nat Methods.

[10] Hartwig B, James GV, Konrad K, Schneeberger K, Turck F (2012). Fast isogenic mapping-by-sequencing of ethyl methanesulfonate-induced mutant bulks. Plant Physiol.

[11] Blumenstiel JP, Noll AC, Griffiths JA, Perera AG, Walton KN, Gilliland WD, Hawley RS, Staehling-Hampton K. Identification of EMS-induced mutations in Drosophila melanogaster by whole-genome sequencing. Genetics. 2009;182(1):25–32. 10.1534/genetics.109.101998 PMC2674820 19307605

[12] Austin RS, Vidaurre D, Stamatiou G, Breit R, Provart NJ, Bonetta D, Zhang J, Fung P, Gong Y, Wang PW, et al. Next-generation mapping of arabidopsis genes. Plant J. 2011;67:715–25. 10.1111/j.1365-313X.2011.04619.x 21518053

[13] Abe A, Kosugi S, Yoshida K, Natsume S, Takagi H, Kanzaki H, Matsumura H, Mitsuoka C, Tamiru M, Innan H, et al. Genome sequencing reveals agronomically important loci in rice using MutMap. Nat Biotechnol. 2012;30(2):174–8. 10.1038/nbt.2095 22267009

[14] Beissinger TM, Hirsch CN, Vaillancourt B, Deshpande S, Barry K, Buell CR, Kaeppler SM, Gianola D, de Leon N. A genome-wide scan for evidence of selection in a maize population under long-term artificial selection for ear number. Genetics. 2014;196(3):829–40. 10.1534/genetics.113.160655 PMC3948809 24381334

[15] Yang J, Jiang H, Yeh CT, Yu J, Jeddeloh JA, Nettleton D, Schnable PS. Extreme-phenotype genome-wide association study (XP-GWAS): a method for identifying trait-associated variants by sequencing pools of individuals selected from a diversity panel. Plant J. 2015;84(3):587–96. 10.1111/tpj.13029 26386250

[16] Varshney RK, Terauchi R, McCouch SR (2014). Harvesting the promising fruits of genomics: applying genome sequencing technologies to crop breeding. PLoS Biol.

[17] Nordborg M, Tavaré S (2002). Linkage disequilibrium: what history has to tell us. Trends Genet.

[18] Dohm JC, Minoche AE, Holtgrawe D, Capella-Gutierrez S, Zakrzewski F, Tafer H, Rupp O, Sorensen TR, Stracke R, Reinhardt R. The genome of the recently domesticated crop plant sugar beet (Beta vulgaris). Nature. 2014;505(7484):546–9. 10.1038/nature12817 24352233

[19] Minoche AE, Dohm JC, Schneider J, Holtgräwe D, Viehöver P, Montfort M, Sörensen TR, Weisshaar B, Himmelbauer H. Exploiting single-molecule transcript sequencing for eukaryotic gene prediction. Genome Biol. 2015;16:184. 10.1186/s13059-015-0729-7 PMC4556409 26328666

[20] Chung HH, Schwinn KE, Ngo HM, Lewis DH, Massey B, Calcott KE, Crowhurst R, Joyce DC, Gould KS, Davies KM, et al. Characterisation of betalain biosynthesis in Parakeelya flowers identifies the key biosynthetic gene DOD as belonging to an expanded LigB gene family that is conserved in betalain-producing species. Front Plant Sci. 2015;6:499. 10.3389/fpls.2015.00499 PMC4493658 26217353

[21] Clement JS, Mabry TJ (1996). Pigment evolution in the Caryophyllales: a Systematic Overview. Bot Acta.

[22] Keller W (1936). Inheritance of some major color types in beets. J Agric Res.

[23] Goldman IL, Austin D (2000). Linkage among the R, Y and BI loci in table beet. Theor Appl Genet.

[24] Hatlestad GJ, Sunnadeniya RM, Akhavan NA, Gonzalez A, Goldman IL, McGrath JM, Lloyd AM. The beet R locus encodes a new cytochrome P450 required for red betalain production. Nat Genet. 2012;44(7):816–20. 10.1038/ng.2297 22660548

[25] Hatlestad GJ, Akhavan NA, Sunnadeniya RM, Elam L, Cargile S, Hembd A, Gonzalez A, McGrath JM, Lloyd AM. The beet Y locus encodes an anthocyanin MYB-like protein that activates the betalain red pigment pathway. Nat Genet. 2015;47(1):92–6. 10.1038/ng.3163 25436858

[26] Magwene PM, Willis JH, Kelly JK (2011). The statistics of bulk segregant analysis using next generation sequencing. PLoS Comput Biol.

[27] DePristo MA, Banks E, Poplin R, Garimella KV, Maguire JR, Hartl C, Philippakis AA, del Angel G, Rivas MA, Hanna M, et al. A framework for variation discovery and genotyping using next-generation DNA sequencing data. Nat Genet. 2011;43(5):491–8. 10.1038/ng.806 PMC3083463 21478889

[28] Cingolani P, Platts A, le Wang L, Coon M, Nguyen T, Wang L, Land SJ, Lu X, Ruden DM. A program for annotating and predicting the effects of single nucleotide polymorphisms, SnpEff: SNPs in the genome of Drosophila melanogaster strain w1118; iso-2; iso-3. Fly (Austin). 2012;6(2):80–92. 10.4161/fly.19695 PMC3679285 22728672

[29] Sims D, Sudbery I, Ilott NE, Heger A, Ponting CP (2014). Sequencing depth and coverage: key considerations in genomic analyses. Nat Rev Genet.

[30] Laitinen RA, Schneeberger K, Jelly NS, Ossowski S, Weigel D (2010). Identification of a spontaneous frame shift mutation in a non-reference Arabidopsis thaliana accession using whole genome sequencing. Plant Physiol.

[31] McKenna A, Hanna M, Banks E, Sivachenko A, Cibulskis K, Kernytsky A, Garimella K, Altshuler D, Gabriel S, Daly M, et al. The genome analysis toolkit: a MapReduce framework for analyzing next-generation DNA sequencing data. Genome Res. 2010;20(9):1297–303. 10.1101/gr.107524.110 PMC2928508 20644199

[32] Bolger AM, Lohse M, Usadel B (2014). Trimmomatic: a flexible trimmer for Illumina sequence data. Bioinformatics.

[33] FastQC, a quality control tool for high throughput sequence data. [http://www.bioinformatics.babraham.ac.uk/projects/fastqc/]. Accessed 1 Feb 2013.

[34] Li H, Durbin R (2009). Fast and accurate short read alignment with Burrows-Wheeler transform. Bioinformatics.

[35] Li H, Handsaker B, Wysoker A, Fennell T, Ruan J, Homer N, Marth G, Abecasis G, Durbin R. The sequence alignment/map format and SAMtools. Bioinformatics. 2009;25(16):2078–9. 10.1093/bioinformatics/btp352 PMC2723002 19505943

[36] A set of Java command line tools for manipulating high-throughput sequencing data (HTS) data and formats. [http://broadinstitute.github.io/picard/]. Accessed 21 Oct 2013.

[37] Van der Auwera GA, Carneiro MO, Hartl C, Poplin R, Del Angel G, Levy-Moonshine A, Jordan T, Shakir K, Roazen D, Thibault J, et al. From FastQ data to high confidence variant calls: the Genome Analysis Toolkit best practices pipeline. Current Protocols in Bioinformatics. 2013;11(1110):11.10.1–11.10.33. 10.1002/0471250953.bi1110s43 PMC4243306 25431634

